# Characterization and Biodegradability of Rice Husk-Filled Polymer Composites

**DOI:** 10.3390/polym13010104

**Published:** 2020-12-29

**Authors:** Saw Yin Yap, Srimala Sreekantan, Mohd Hassan, Kumar Sudesh, Ming Thong Ong

**Affiliations:** 1 School of Materials and Mineral Resources Engineering, Universiti Sains Malaysia, Nibong Tebal 14300, Penang, Malaysia; yapsawyin@hotmail.com (S.Y.Y.); mohdhassan43@gmail.com (M.H.); 2 School of Biological Sciences, Universiti Sains Malaysia, Gelugor 11800, Penang, Malaysia; ksudesh@usm.my; 3 Institute for Research in Molecular Medicine (INFORMM), Universiti Sains Malaysia, Gelugor 11800, Penang, Malaysia; omt@usm.my

**Keywords:** rice husk waste, polybutylenes adipate-Co-terephthalate (PBAT), polybutylene succinate (PBS)

## Abstract

The fabrication of affordable biodegradable plastics remains a challenging issue for both the scientific community and industries as mechanical properties and biodegradability improve at the expense of the high cost of the material. Hence, the present work deals with fabrication and characterization of biodegradable polymer with 40% rice husk waste filler and 60% polymer-containing mixture of polybutylene succinate (PBS) and poly butylenes adipate-Co-terephthalate (PBAT) to achieve good mechanical properties, 92% biodegradation in six months, and competitive pricing. The challenge in incorporating high amounts of hydrophilic nature filler material into hydrophobic PBS/PBAT was addressed by adding plasticizers such as glycerol and calcium stearate. The compatibilizers such as maleic anhydride (MA) and dicumyl peroxide (DCP) was used to improve the miscibility between hydrophobic PBS/PBAT and hydrophilic filler material. The component with the formulation of 24:36:40 (PBS/PBAT/TPRH) possessed the tensile strength of 14.27 MPa, modulus of 200.43 MPa, and elongation at break of 12.99%, which was suitable for the production of molded products such as a tray, lunch box, and straw. The obtained composite polymer achieved 92% mass loss after six months of soil burial test confirming its biodegradability.

## 1. Introduction

Petroleum-based plastics have been extensively used in numerous fields such as packaging bags, consumer goods, medical equipment, the automotive sector, and construction sites. The global production of plastics was valued at around 52.9 million tons in 2017. Asia commanded up to 31.4% of the global market in 2018, with a value of 16.61 million tons [1]. The World Wide Fund for Nature (WWF) reported that Malaysia was one of the top plastic consumers in Asia, with 16.8 kg of plastic consumption per person reported annually. In 2020, the consumption of plastic in Malaysia was 543.5 kilo tons [2]. Polyolefin plastics dominate 35 to 45 percent of the synthetic polymer produced in total [3]. The heavy usage of plastics produces a hefty amount of non-degradable wastes, which induces harmful effects on the ecosystem. The environmental pollution incurred due to the use of these traditional polymers has introduced the development of biodegradable polymers.

Biodegradable polymers such as polylactic acid (PLA) [4], polycaprolactone (PCL) [5], poly (3-hydroxybutyrate-co-3-hydroxyvalerate) (PHBV) [6], polybutylene succinate (PBS) [7], and polybutylene adipate-co-terephthalate (PBAT) [8] were used by researchers to obtain cost-effective biocomposites with superior properties. PBS is considered as one of the promising alternatives because of its virtues in strength, toughness, excellent biodegradability, and good processing parameters [9]. Nevertheless, PBS shows insufficient impact strength and gas barrier issues for certain applications. This problem can be addressed by the physical blending of PBS with highly flexible PBAT [9]. PBAT is a 100% biodegradable polymer exhibiting good thermal and mechanical properties [10]. The tensile strength is comparable with low-density polyethylene [11]. The studies conducted by Muthuraj et al. [12] showed good compatibility was achievable in the PBS/PBAT (40/60 wt%) blends, which was further proved by even dispersion of the PBS phase in the PBAT phase. However, poor cost performance existed when a comparison was done with a polymer like polyethylene (PE) and polypropylene (PP), thus restricting its wide-scale applications for practical usage. Hence in this work, high loading of waste material as fillers was used to reduce the cost of the production of biodegradable plastic. Blending with biodegradable materials was considered as an effective strategy to overcome the costs incurred during the material processing.

Natural fibers possess economic advantages in comparison with synthetic fibers. Besides this, they are lightweight, renewable, and biodegradable. One of the most commonly used biodegradable materials is starch. It is regarded as the optimal additive due to its cheap cost and availability. It also derives many advantages, such as renewability and complete biodegradability from nature [13,14]. However, starch is a hydrophilic polymer, while PBS and PBAT are hydrophobic polymers. Hydrophilic starch and hydrophobic polyesters are thermodynamically incompatible, having improper adhesion characteristics [15]. These properties can be enhanced by adding a plasticizer in starch to generate thermoplastic starch (TPS) [16]. The plasticizers such as water, glycerol, and polyvinyl alcohol are used to generate thermoplastic starch [15]. Considering this fact, the high tensile properties and thermal characteristics of the blend can be attained if TPS is well dispersed in PBS/PBAT matrix, hence possessing good phase interaction between TPS and PBS/PBAT [17].

Previous researchers have reported the potential of using waste material as filler in the composite fabrication [18,19,20]. Hence, rice husk waste was utilized in this study for the fabrication of a biodegradable polymer. It was reported that total rice production worldwide in 2018/2019 was valued around 495.9 metric tons [21]. The rice production approximately generates 123.87 metric tons of rice husk; out of that, some proportion is used for cattle feeding, while the remaining is dumped as waste in landfill and later burned openly. Burning of rice husk contributes to high CO_2_ emission and environmental pollution, which further causes health issues [22]. Hence, utilization of this renewable agriculture waste material to form biodegradable polymers would resolve environmental issues, and hence contribute towards Sustainable Development Goal 12, which ensures sustainable consumption and production patterns. It could also be a way to resolve cost and waste disposal issues.

Several reported works on the utilization of rice husks in polymers are summarized in Table 1. As seen, the rice husk has been dominantly utilized in polyolefin rather than in bioplastics polymers. For bioplastic polymers, filler loading up to 5 and 30% have been utilized with PLA and PBAT, respectively. The PBAT/RH with a 70:30 weight ratio exhibited tensile strength, Young’s modulus, and elongation at break as 14.5 MPa, 54 MPa, and 820%, respectively [19]. It possessed lower tensile strength and Young’s modulus than PP/RH 70:30 wt%. Thus, more rice husk loading is required to achieve better tensile strength and Young’s modulus. Hydrophilic rice husk and hydrophobic PBAT and PBS are thermodynamically incompatible, having poor miscibility. Therefore, in this work, rice husk waste (up to 40%) was used after modifying it with glycerol to form thermoplastic rice husk (TPRH). The outcome of 40% loading of TPRH with PBS/PBAT is investigated in this study.

The bare PBAT was soft, and therefore, blending with PBS to mold a product with a stiffness of 78.13 MPa [29] was an essential step. However, blending filler and polymers at nearly equal proportions resulted in an immiscibility issue. Hence, compatibilizer is required to reduce the interfacial tension and to form a co-continuous structure [30]. Therefore, in this work, compatibilizer such as MA and DCP was used to avoid phase separation and to promote an excellent interfacial adhesion for improved mechanical properties of PBS/PBAT with TPRH. The results of TPRH samples were compared with PBS/PBAT filled with TPS.

## 2. Materials and Methods

### 2.1. Materials

The corn starch (particle size: 14.89 µm) was provided by Thye Huat Chan Sdn Bhd (Penang, Malaysia). Rice husk waste (particle size: 16.59 µm) was obtained from Fragstar Corporation Sdn. Bhd. (Kedah, Malaysia). The polymer poly (butylene adipate co-terephthalate) (PBAT) with T_m_ = 115–125 °C and MRF (190 °C/2.16 kg):3.0–5.0 g/10 min) was purchased from ZhuHai WanGo Chemical Co., Ltd. (Zhuhai, China) under the commercial name of A400. Polybutylene succinate (PBS; T_m_: 120 °C; MRF: 10–15g/10 min) (injection molding grade) was obtained from Hefei TNJ Chemical Industry Co., Ltd. (Hefei, China). Glycerol was obtained from Aldrich Chemical Company (St. Louis, MO, USA) with a purity of 85%. Calcium stearate was purchased from Strem Chemical Company (Massachusetts, United States). The reactive modification of PBAT/PBS was performed using maleic anhydride (98% pure), which was purchased from Fluka (Jawa Timur, Indonesia). Dicumyl peroxide with a purity of 99% obtained from Sigma-Aldrich (St. Louis, MO, USA), was used as an initiator.

### 2.2. Polymer Composite Preparation

Starch, rice husk, PBS, and PBAT were dried at 70 °C overnight before use. TPS was prepared using a Haake internal mixer at 100 °C with a rotor speed of 50 rpm by mixing 75 wt% starch with 23 wt% glycerol. TPRH was also prepared by mixing 90.0 wt% rice husk with 9.1 wt% glycerol using a Haake internal mixer at 100 °C with a rotor speed of 50 rpm. The PBS, PBAT, maleic anhydride (MA), dicumyl peroxide (DCP), calcium stearate, and TPRH/TPS (with the amount shown in Table 2) were physically mixed in the Haake internal mixer at 160 °C with a rotor speed of 50 rpm for 15 min. Subsequently, the samples were fabricated by a compression molding machine at 160 °C for 6 min preheating time, 3 min heating time, and 2 min cooling time into 140 mm × 180 mm × 0.5 mm sheets. The compositions of the melt-blended specimens are summarized in Table 2.

### 2.3. Material Characterization

#### 2.3.1. Fourier Transforms Infrared Spectroscopy (FT-IR)

Vector-33 FTIR spectrometer (Bruker, Germany) was used to carry out FT-IR measurement to analyze the reaction between PBS, PBAT, and maleic anhydride. The scanning range varied from 4000 to 500 cm^−1^ with a resolution of 16 cm^−1^.

#### 2.3.2. Mechanical Properties

Tensile testing was conducted with a speed of 5 mm^−1,^ according to ASTM D638. The samples were injection molded with standard Type V samples with a thickness of 0.5 mm. The samples were sealed packed in the plastic bags after molding and conditioned at room temperature for 24 h before testing. Five samples were tested for each composition, and the average value with the standard deviation was recorded.

#### 2.3.3. Thermal Behavior

The crystallization kinetics were investigated using a Perkin-Elmer DSC 7 system. Samples were heated from 30 to 200 °C at 10 °C/min. The samples were kept in the molten state for 5 min to remove thermal history and then cooled down to 30 °C at the rate of 10 °C/min. Then, the samples were heated back again to 200 °C at a rate of 10 °C/min to analyze the crystallization characteristic after heating. The percent crystallinity of the PBS and PBAT was calculated using Equation (1).

(1) $$Xc=(\frac{\Delta H_{m}}{\Delta H_{m100}\left(1-w_{P}\right)})\;\times \;100\;\%\;$$

where ∆*H_m_*_100_ is the theoretical enthalpy of melting for 100% crystalline PBS (110.3 J/g) and PBAT (114 J/g), w_p_ is the weight fraction of the PBS, TPS, and TPRH in the PBS/PBAT blend.

#### 2.3.4. Morphology Analysis

The morphology of the fractured specimen was observed using scanning electron microscopy (SEM) operated at an acceleration voltage of 5 kV. The samples were vacuum coated with a thin layer of gold before testing.

#### 2.3.5. Water Absorption Test

The molded samples (size 10 × 10 × 0.5 mm) were immersed in distilled water for a different interval of time at room temperature. For each interval, the samples were gently wiped with soft tissue paper to remove the excess water on the surface. The water absorption (%) was calculated using Equation (2):

(2) $$\mathrm{Water}\mathrm{absorption}\;(\%)=\frac{W_{f}-W_{i}}{W_{f}}$$

where *w_f_* is the weight of the sample after immersion and *w_i_* is the sample weight before immersion.

#### 2.3.6. Soil Burial Test

Compost soil was collected from agriculture field at the USM engineering campus, Malaysia for soil burial test. Molded samples (size 10 × 10 × 0.1 mm) were buried at a depth of 15 cm in the ground in USM engineering campus. Five specimens of each sample were taken out in 2, 4, and 6 months for testing. Then, the samples were rinsed with distilled water and blotted with tissue paper to remove dirt. The samples were dried until a constant weight was achieved. The percentage of weight loss was calculated using Equation (3):

(3) $$\%\mathrm{of}\mathrm{weight}\mathrm{loss}=\frac{W_{X}-W_{O}}{W_{O}}\;\times \;100\;\%$$

where *W_x_* and *W_o_* indicate the weights of the collected samples and initial weight of the samples.

## 3. Results

### 3.1. FT-IR Analysis

**Figure 1:**
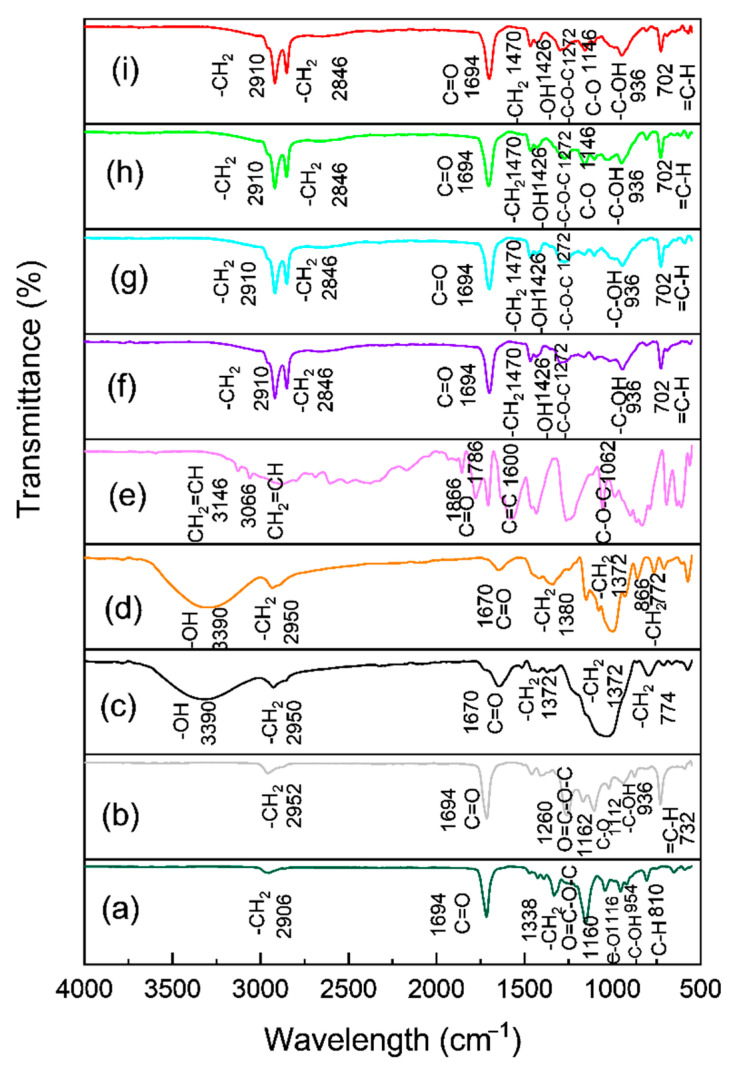
FT-IR spectra of the PBS/PBAT/TPS composites and PBS/PBAT/TPRH composites (**a**) PBS, (**b**) PBAT, (**c**) rice husk, (**d**) starch, (**e**) maleic anhydride, (**f**) TPRH48/12, (**g**) TPS48/12, (**h**) TPRH36/24, and (**i**) TPS36/24.

The performance of the grafting process within PBS/PBAT/TPS and PBS/PBAT/TPRH was evaluated through the FT-IR technique, and the results are shown in Figure 1. For comparison, FT-IR results of bare PBS and bare PBAT are also displayed in Figure 1. Figure 1a displays a peak at 1160 cm^−1^, which belonged to the aliphatic ester groups of PBS samples, confirming its aliphatic structure [31]. The bands at the 810, 964, 1116, and 1694 cm^−1^ confirmed the C-H bending of alkane, -C-OH blending of carboxylic acids group, -C-O- stretching vibrations, and C = O stretching vibration in the ester linkages of PBS [32,33]. The band at 1338 cm^−1^ and 2906 cm^−1^ were attributed to the symmetric and asymmetric deformational vibrations of -CH_2_- groups of the PBS structure [34]. Figure 1b exhibits the peaks of bare PBAT at 1260 and 1162 cm^−1^, which were ascribed to O = C-O-C stretching of aromatic and aliphatic ester groups, respectively, validating its aliphatic-aromatic structure. The peaks at 732, 936, and 1112 cm^−1^ are attributed to = C-H bending of benzene ring, -C-OH blending of carboxylic acids group, and -C-O- stretching vibrations of PBAT [32,35]. The band at 1694 and 2952 cm^−1^ are attributed to the C = O stretching vibration and -CH_2_- groups of PBAT, respectively.

Figure 1c,d represent the FT-IR spectra of rice husk and starch. The broad absorption band at 3390 cm^−1^ was ascribed to the stretching occurring in the -OH group [36,37]. The band indicated rice husk and starch had a considerable amount of surface absorbed moisture [38]. The peak at 2950 cm^−1^ was assigned to C-H stretching vibration. The presence of a band at 1670 cm^−1^ affirmed the stretching vibration of the C = O group in rice husk and starch. The absorption band at 1372 cm^−1^ was attributed to -CH_2_ scissoring vibrations [39,40]. The peak around 774 cm^−1^ showed the presence of -CH_2_ blending [41]. Figure 1e displays the FT-IR spectra of maleic anhydride. The absorption bands at 3146, 3066, 1600, and 1062 cm^−1^ were assigned to asymmetrical C-H stretching vibration (CH_2_ = CH_2_), symmetrical C-H stretching vibration (CH_2_ = CH_2_), C = C stretching band, and C-O-C symmetrical stretching band, respectively [42]. The peaks at 1866 cm^−1^ and 1786 cm^−1^ were assigned to the C = O stretching vibration of maleic anhydride [43].

Figure 1f–i show the FT-IR spectra of TPRH48/12, TPS48/12, TPRH36/24, and TPS36/24 blends, respectively. The spectra show a similar peak for bare PBS and PBAT. However, an additional band at 2846 cm^−1^ suggests the -CH_2_ group from the TPRH or TPS and CH_2_ = CH vibration in the cyclic MA. Since the MA was only applied in 2 p/hr, which is considered a small amount. Thus, this bond was corresponded to the -CH_2_ group from the TPRH or TPS. This confirms the reaction between PBS/PBAT and TPRH or TPS and addresses the incompatibility between polymer matrix and filler material [44]. In summary, Figure 1 shows an insignificant difference between PBS/PBAT/TPRH and PBS/PBAT/TPS blends as rice husk and starch are organic-based fillers having similar functional groups. This indicated the potential of using waste material such as rice husk to replace starch in biodegradable plastics. The presence of strong absorption bands at 1694 and 1470 cm^−1^ is associated with the stretching vibration of the C = O group and -CH_2_ scissoring vibrations of TPRH and TPS, respectively [37]. Besides, the band at 1426 cm^−1^ confirmed the -OH group of glycerol, which was used to modify the surface of rice husk and starch to form TPRH and TPS [33].

### 3.2. Mechanism between MA, DCP, PBS, PBAT, and TPRH/TPS

**Figure 2:**
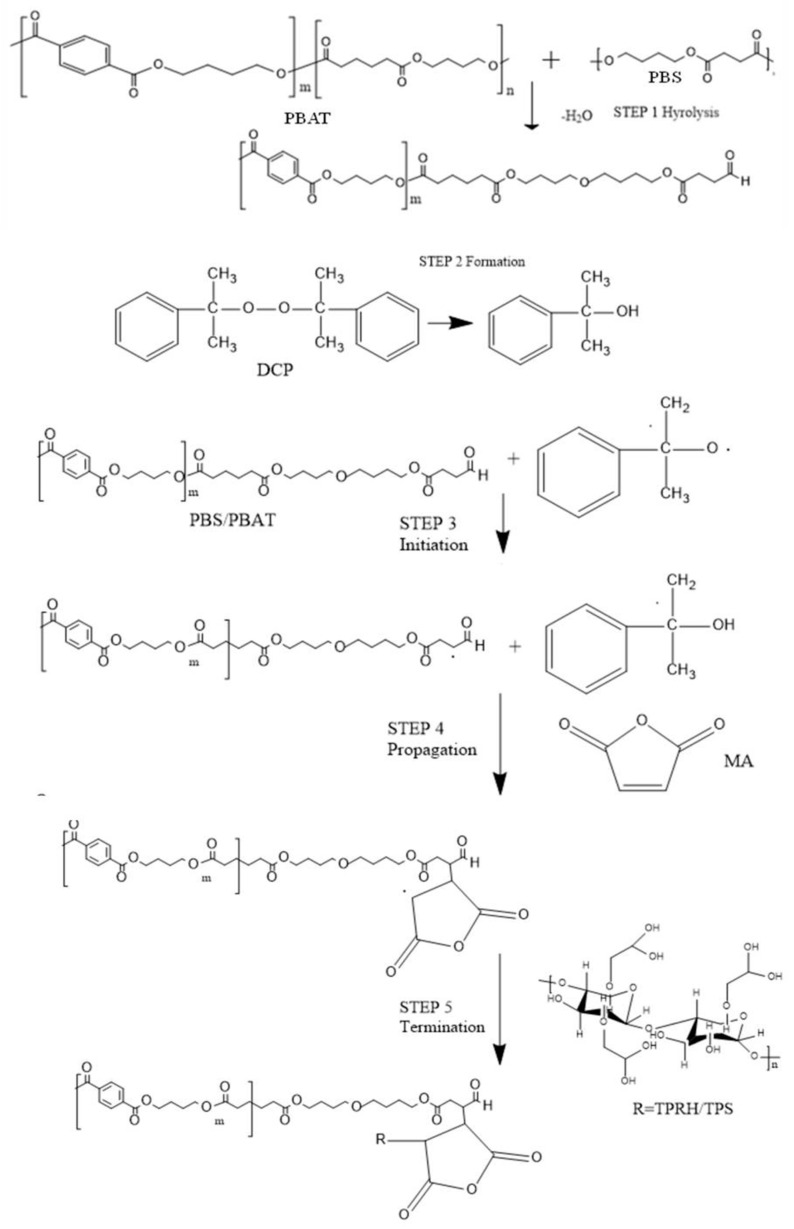
The mechanism between maleic anhydride, DCP, PBS, PBAT, and TPRH/TPS.

Figure 2 presents the mechanism between MA, DCP, PBS, PBAT, and TPRH/TPS. The reaction between PBS and PBAT was formed via the hydrolysis reaction and forming the C-O bond. DCP decomposed at the initial step to form primary radicals. These primary radicals attracted the hydrogen atom from the PBS/PBAT backbone and yielded PBS/PBAT radicals at the initiation step. The propagation step shows the maleic anhydride (MA) molecules grafted onto the PBS/PBAT radicals to form PBS/PBAT-MA radicals and followed by termination reaction. The PBS/PBAT-MA radicals might undergo hydrogen transfer from TPS and TPRH and form the final product. The termination step showed the reaction between PBS/PBAT-g-MA with TPS and TPRH.

### 3.3. Mechanical Properties

**Figure 3:**
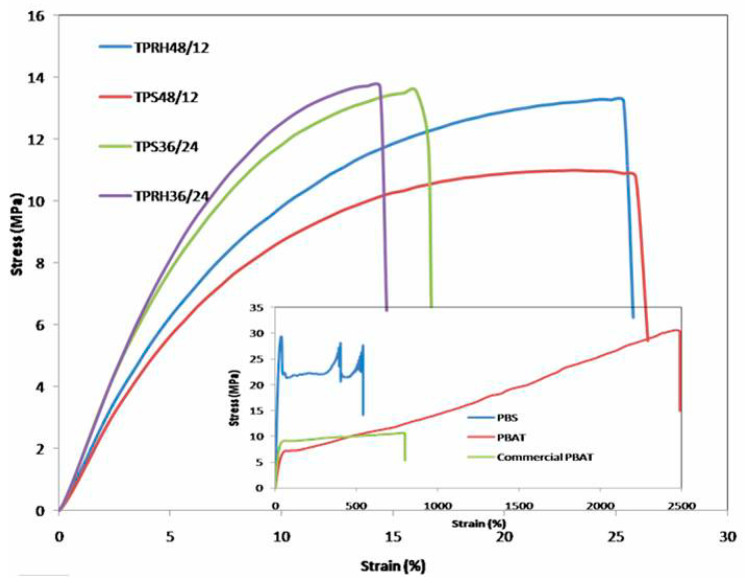
Stress–strain curves of PBS/PBAT/TPRH and PBS/PBAT/TPS composites.

Typical stress–strain curves for bare PBS, bare PBAT, PBS/PBAT/TPRH, and PBS/PBAT/TPS composites are shown in Figure 3. Bare PBS, bare PBAT, and commercial PBAT are highly elastic polymers with high elongation at break. With the introduction of 40% TPRH or TPS, a significant change for both tensile strength and elongation at break was observed compared to bare PBS and bare PBAT. Table 3 represents the tensile data obtained from TPRH composites and TPS composites at a different ratio. For the testing, both TPRH and TPS (as filler materials) were fixed at 40% by weight. The tensile strength, Young’s modulus, and elongation at break of bare PBAT were found to be 38.99 MPa, 16.1 MPa, and 1421.9%, respectively. The bare PBS possessed lower tensile strength (30.63 MPa), lower elongation at break (547.45%), and higher Young’s modulus (166.23 MPa) when compared with PBAT. The higher incorporation of PBS caused an increment in tensile strength and Young’s modulus, but decrement in elongation at break. The increment of Young’s modulus and decrement in elongation at break is because of PBS, which has a lower elongation at break (547.45%) and higher Young’s modulus 166.23 MPa. The TPRH36/24 and TPS36/24 were prepared using a PBAT:PBS ratio of 36:24. Both the composites with more incorporation of PBS exhibited better tensile strength and Young’s modulus, but lower elongation at break when compared with TPRH48/12 and TPS48/12, which is consistent with the work of Boonprasertpoh et al. [29]. This shows that when both polymers (PBS and PBAT) were at a comparable amount, the co-continuous phase occured. This was confirmed with SEM morphology, which is elaborated in the later section. Furthermore, the DSC result shows that TPRH36/24 and TPS36/24 have higher relative crystallinity than TPRH48/12 and TPS48/12, supporting PBS contribution towards a crystallization process. This subsequently affects the tensile strength of the polymer matrix.

Table 3 reveals that both TPRH and TPS composites showed lower tensile strength, elongation at break, but higher Young’s modulus when compared with bare PBS and PBAT. This result was in agreement with the study of Hardinnawirda and Aisha [45], who claimed that when the rice husk loading exceeds 15 wt%, the tensile strength shows a remarkable decrement. Incorporation of 40% TPS or TPRH caused linear decrement in the tensile strength and elongation at break of PBS/PBAT matrix, which was same as the results of Garalde et al. [46]. The decrement of tensile strength was due to the stiffness of TPS or TPRH, causing the steric hindrance effect ascribed to cross-linked aromatic structures of PBS and PBAT. The presence of filler material caused reinforcing effects on the properties of the composites, thus reducing the mobility of polymer chains. The improved Young’s modulus compared to bare PBAT was due to the enhanced interaction between the carbonyl group of PBS/PBAT matrix and OH groups of TPRH or TPS. The enhanced interaction allows efficient stress transfer from semi-crystalline TPRH to PBS/PBAT [46].

PBAT/PBS/TPRH composite blends with 40% filler prepared in this work, attained remarkable mechanical properties when compared to reported work by Sabetzadeh and his colleagues with just 15% loading of starch filler [47]. The tensile strength and elongation at break of the 15% filler in LDPE were reported to be between 9–12 MPa and 260–360%, respectively [47]. The PBAT/PBS/TPRH composites prepared in this work exhibited better tensile strength and Young’s modulus but lower elongation at break than commercial PBAT. For the injection molding process, tensile strength, Young’s modulus, and elongation at break required are 11.70 MPa [29], 78.13 MPa [29] and 9% [48]. Thus, all the samples prepared in this work are applicable for the injection molding process as they possess the required mechanical properties.

### 3.4. Thermal Behavior

**Figure 4:**
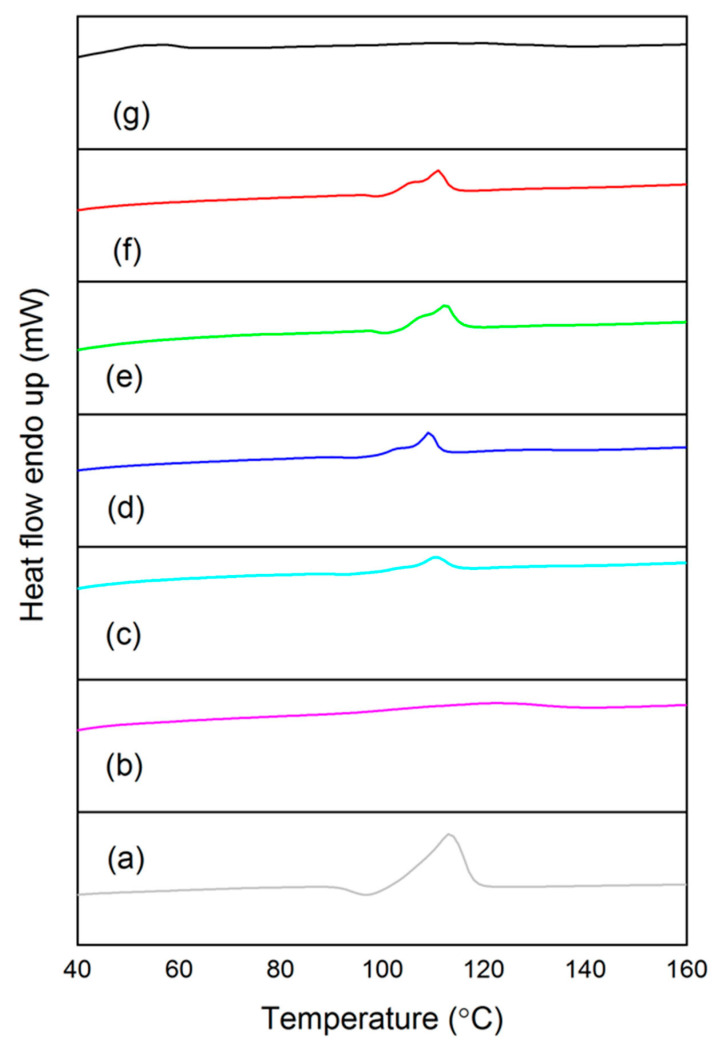
Second-heating Differential Scanning Calorimetry curves of (**a**) PBS, (**b**) PBAT, (**c**) TPRH48/12, (**d**) TPS48/12, (**e**) TPRH36/24, (**f**) TPS36/24, and (**g**) commercial PBAT.

Table 4 summarizes the melting point, enthalpy of melting at 100% crystallinity, and crystallinity of the compounded blends. Figure 4 shows that all samples possessed endothermic peaks. The bare PBAT displayed a broad peak at 121.68 °C, while bare PBS exhibited a sharp peak at 114.04 °C. The direct proof of polymer miscibility was obtained by observing the change in the melting point (T_m_) of both polymers in the blends. One melting point endotherm was observed for TPRH48/12, TPS48/12, TPRH36/24, and TPS36/24. This scenario indicated that PBS and PBAT were miscible. The T_m_ of rice husk and starch was absent because the melting point was beyond 200 °C [49,50]. The melting point of corn starch and rice husk is 256 °C–258 °C [50] and 1440 °C, respectively [49].

The bare PBS with a high degree of crystallinity (64.03%) was less susceptible to water absorption because of smaller gaps present between the polymer chains. It was evident from the test results that the addition of filler material slightly reduced the melting point of PBS/PBAT blends. The composites TPRH48/12, TPS48/12, TPRH36/24, and TPS36/24 showed a lower degree of crystallization than PBS. This was due to fillers in polymer matrix reduces the mobility of polymer chains, thus causing the steric hindrance effect ascribed to the cross-linked aromatic structure, leading to a reduction in the extent of crystallinity [51].

Relatively, the composites TPRH36/24 and TPS36/24 exhibited a higher degree of crystallization than TPRH48/12 and TPS48/12. This was due to the high amount of PBS, which possesses higher crystallinity. This phenomenon was consistent with the results of the tensile test, whereby TPRH36/24 and TPS36/24 yield higher tensile strength, as shown in Table 3. However, there was no significant difference found in crystallinity between PBS/PBAT/TPRH blends and PBS/PBAT/TPS blends, indicating the potential of using rice husk waste to substitute starch in the polymer matrix.

### 3.5. Morphology Analysis

**Figure 5:**
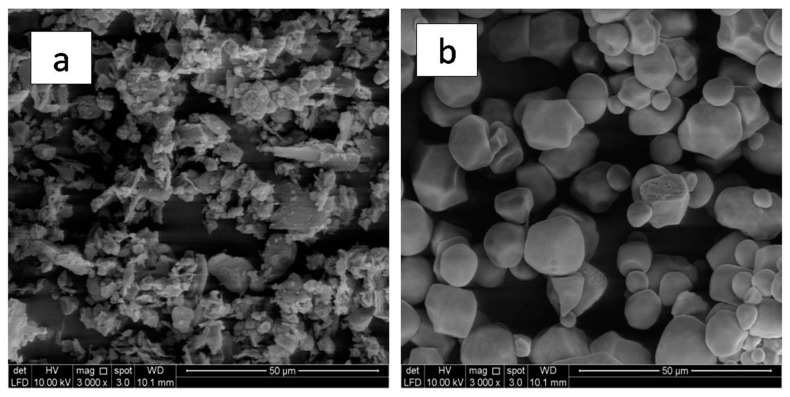
Surface morphology of (**a**) irregular TPRH granules and (**b**) spherical TPS granules.

**Figure 6:**
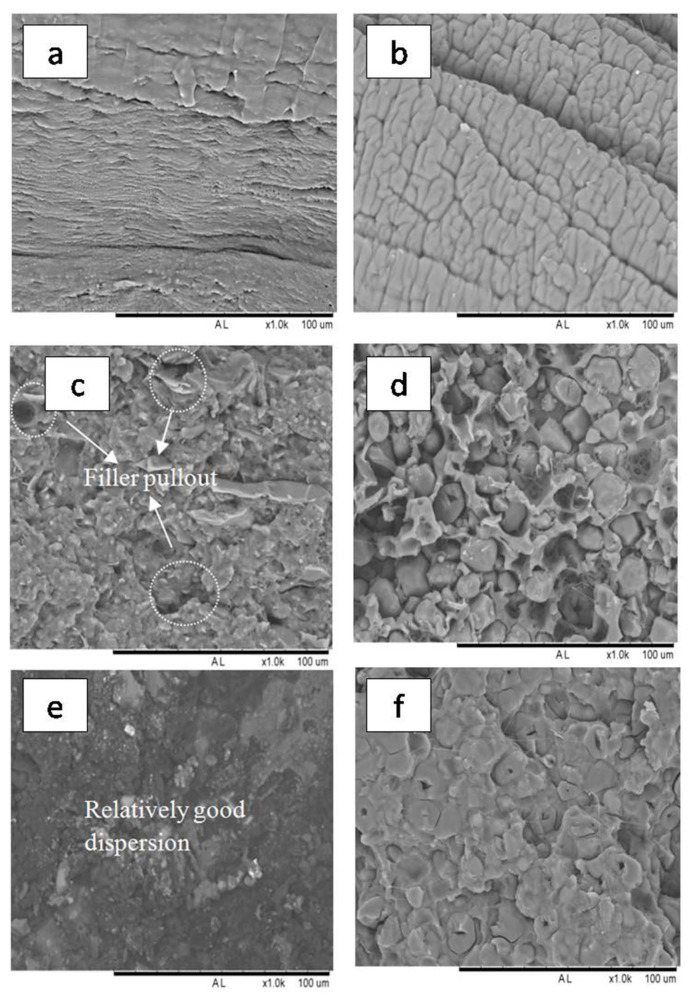
Fractured morphology of (**a**) PBS, (**b**) PBAT, (**c**) TPRH48/12, (**d**) TPS48/12, (**e**) TPRH36/24, and (**f**) TPS36/24.

Figure 5 shows the surface morphology of TPRH and TPS with irregular (Figure 5a) and spherical (Figure 5b) shape, respectively. The TPS granules with spherical shape caused a reduction in the contact area of the polymer matrix when compared with the irregular shape of TPRH [52]. For bare polymers of PBS (Figure 6a) and PBAT (Figure 6b), smooth surface morphology was observed while with filler loading, a rough surface was observed as shown in Figure 6c–f. When 40% of TPRH was added into PBAT/PBS matrix, the TPRH particles dispersed homogeneously in the PBAT/PBS matrix, as shown in Figure 6c,e. Some voids in the interfacial boundary were observed in TPRH48/12 due to the pull out of the rice husk particle from the PBAT/PBS matrix. This is caused by the difference in polarity between the hydrophilic rice husk and the hydrophobic PBAT. Fewer filler pullouts with good fibers-matrix adhesion were observed in TPRH36/24 (Figure 6e) as compared to TPRH48/12 (Figure 6c). For TPS blends, a similar trend was observed in TPS48/12 (Figure 6d), which showed a higher filler pullout than the TPS36/24 (Figure 6f). The SEM morphology of TPRH36/24 and TPS36/24 exhibited the co-continuous phase as PBS and PBAT are at a comparable amount. Hence, this explains the less pull out circumstances and better tensile strength in TPRH36/24 and TPS36/24 in comparison to TPRH48/12 and TPS48/12.

### 3.6. Water Absorption

**Figure 7:**
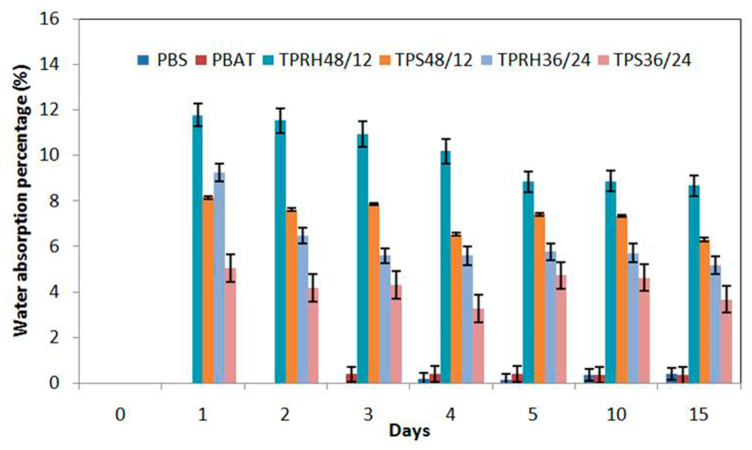
Water absorption of bare PBS, bare PBAT, and PBS/PBAT blends with immersion times.

The water absorption of the bare PBS, PBAT, and PBS/PBAT blends are shown in Figure 7. All polymers undergo three stages during the water absorption which are absorption, saturation, and swelling. The water absorption of PBAT and PBS film increased slightly and achieved saturation of around 0.35% after 10 days of immersion in water. The low water absorption capacity was due to PBAT and PBS being hydrophobic polymers with the presence of acyl groups [31]. However, at the initial stage (day 3), it was obvious that PBS, having higher crystallinity, possessed a water absorption capacity of 0 wt%, while PBAT had a capacity of 0.39%, which indicated that the degree of crystallinity is an important factor for water absorption of the polymer. Besides this, PBS with a high degree of crystallinity was less prone to water absorption as compared to PBAT due to the smaller amorphous region accessible for water intake.

The PBS/PBAT/TPRH composites showed a larger water absorption capacity than bare PBAT and PBS. The water absorption rose tremendously in the first 24 h, and reached the saturation limit after 24 h of immersion. The TPRH48/12 and TPRH36/24 showed water absorption capacity at 11.78 and 9.24%, respectively after 24 h. The composite TPRH36/24 possessed a lower water absorption capacity than TPRH48/12 due to the intrinsic nature of PBS with high crystallinity. Moreover, the water absorption capacity of PBS/PBAT/TPRH was also found to be more than PBS/PBAT/TPS, which is an attribute to the lumen and cell wall of rice husk that provide more room for the water absorption [18]. Besides, the hydrophilic nature of rice husk that favors the intermolecular hydrogen bonding enhanced the water absorption of the film.

The swelling in composites occured due to the presence of internal stresses that prevents polymer matrix from absorbing water [53]. The swelling effect was observed for both TPRH and TPS blends after 3 days of immersion, which in agreement with the work was reported by Muthuraj et al. [54]. Nevertheless, TPRH36/24 and TPRH48/12 showed lower water absorption capacity (5.17 and 8.68%) than the reported LDPE by Sabezadeh et al. [47] with 11% absorption capacity at day 15. Water absorption characteristics influence the water vapor barrier properties of a material. Thus, the results indicated that the fabricated polymer in this work has good water vapor barrier property, which would extend the shelf life of the food.

### 3.7. Soil Burial Test

**Figure 8:**
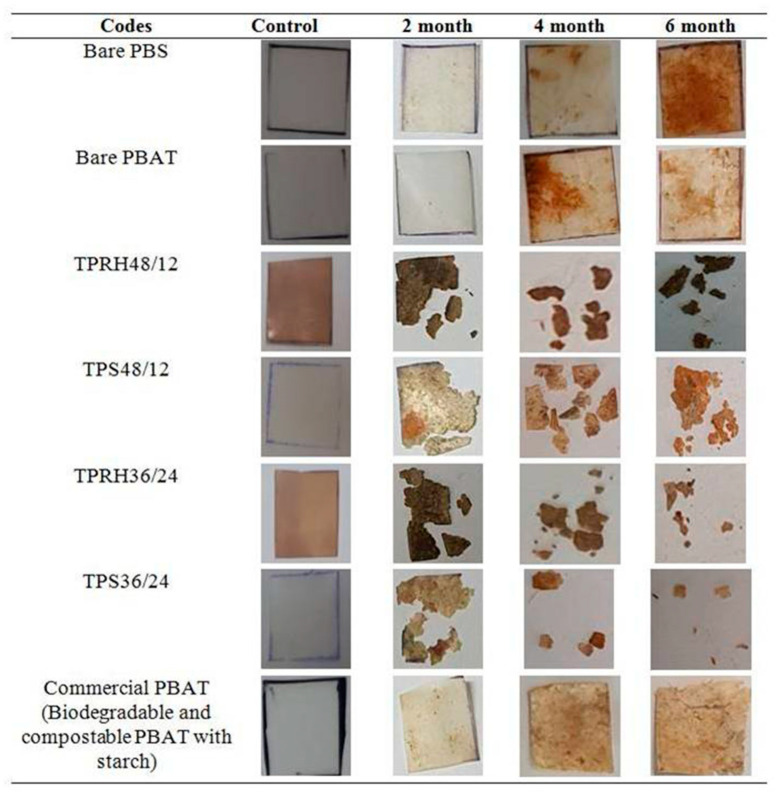
Macroscopic appearance of biodegradation in the soil at different burying times.

The entire composites showed a smoother surface before the degradation process. The mass change in the PBS/PBAT blended composites as a function of degradation time is shown in Table 5. The macroscopic appearance of biodegradation in PBS/PBS blended composites at different burying time is shown in Figure 8. Matting of the sample surface and color change was noticeable after the degradation process. The mass loss percentage increased with increasing burying time for the entire samples, which affirmed the biodegradation properties of PBS, PBAT, and PBS/PBAT blends. Progressive fragmentation was noticed in all the samples as they gradually degraded within 6 months. At the end of 6 months, only a small amount of residual debris remained. The color of the film gradually became brownish with increasing burying time. For TPS36/24, the mass loss percentage reached 97.06% after 6 months, which was in agreement with the results of Dammak et al. [55], indicating a fully biodegradable characteristic of this material. As for TPRH36/24, a similar result was obtained with a maximum mass loss of 92%. TPS48/12 and TPRH48/12 showed a much lower mass loss, indicating higher PBS content expedite the degradation process. The bare PBAT, PBS, and commercial PBAT showed mass loss at 8.9, 9.2, and 32.51%, respectively, after 6 months.

As it was evident from the appearance, PBS/PBAT/TPRH and PBS/PBAT/TPS samples showed faster degradation rates than bare PBAT and PBS. TPS and TPRH are the nutrient source for microorganisms, thus it provides more degradation sites to be attacked by microorganisms [56]. When microorganisms consume the TPS and TPRH, they leave the polymer matrix more porous, which accelerates the biodegradation rate of PBS/PBAT blends [11]. This causes the polymer chains to split into lower molecular weight oligomers, monomers, dimmers, and finally mineralized to CO_2_ and H_2_O [57]. The result also indicated that the utilization of rice husk with a high amount of PBS has the potential to degrade faster and is comparable with TPS (refer to the mass loss for TPRH36/24 and TPS48/12). Although TPS36/24 and TPRH36/24 showed higher crystallinity and lower moisture absorption, they exhibited a higher mass loss percentage than TPS48/12 and TPRH48/12. This is because of the aromatic structure of PBAT, which decreases the mobility of polymer chains, reducing the degradation rate of polymer matrix [31].

**Figure 9:**
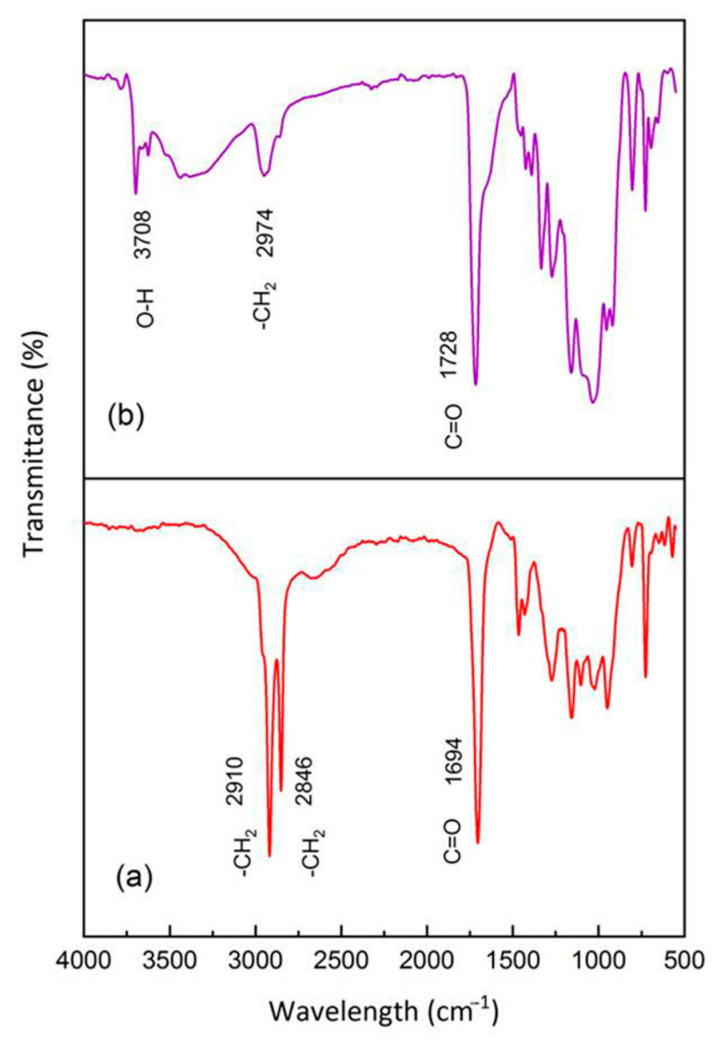
FT-IR spectra of the PBS/PBAT/TPRH composites (**a**) TPRH36/24 before degradation, (**b**) TPRH36/24 after degradation with changes in functional group.

**Figure 10:**
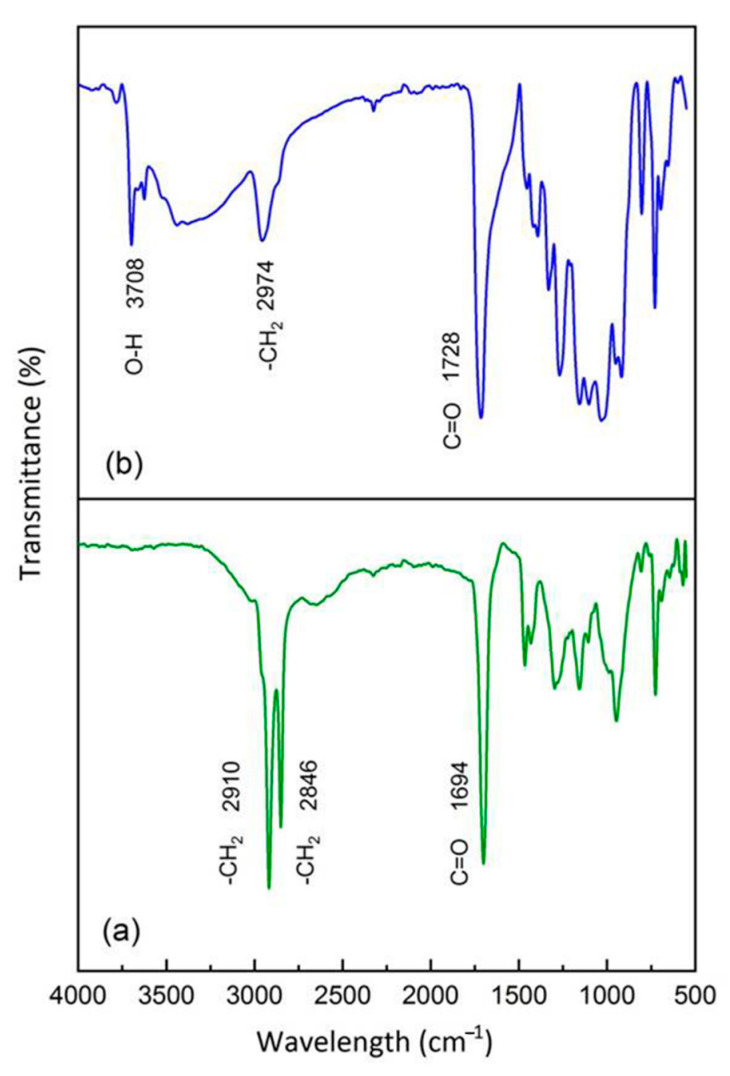
FT-IR spectra of the PBS/PBAT/TPS composites (**a**) TPS36/24 before degradation, (**b**) TPS36/24 after degradation with changes in the functional group.

The sample prone for degradation (TPRH36/24) was selected for FT-IR analysis to observe the changes in the intensity of certain transmittance peak, the formation of new peaks, or migration of the peak position before and after degradation. Figure 9a shows the FT-IR spectra of TPRH36/24 before biodegradation, while Figure 9b depicts FT-IR spectra of TPRH36/24 after 6 months of degradation. It was found that the highly intense -CH_2_ stretching vibration position of the intrinsic polymer diminishes and migrated to 2974 cm^−1^, indicating a significant degradation process of the sample. Moreover, the less intense carbonyl region of the C = O group migrated from 1694 cm^−1^ to a highly intense and broader peak at 1728 cm^−1^, affirming the process of degradation has occurred. The emergence of the new peak was noticed at 3708 cm^−1^ after degradation, which corresponds to the O-H group of absorbed water in the polymer matrix [38]. The disappearance of the -CH_2_ peak at 2846 cm^−1^ indicated the degradation of TPRH and TPS. The changes observed in the FT-IR spectra are in agreement with the polymer oxidation degradation process reported by Celina et al. [58], which showed the dominant carbonyl formation with the diminishing C-H bands. The FTIR spectra of TPS36/24 before and after biodegradation are shown in Figure 10a,b, respectively. The results show that there was no significant difference between TPRH36/24 and TPS36/24 before and after biodegradation. This suggests the potential of using rice husk waste to swap starch in biodegradable polymer composites.

## 4. Conclusions

In this study, it was found that the incorporation of 40% rice husk was able to substitute starch-based biodegradable polymer. Optimization of the ratio PBAT:PBS to 36:24 expedited the biodegradation rate of the samples. PBAT:PBS blends with a 36:24 ratio showed 97.06% mass loss for TPS and 92% for TPRH. A comparable amount of PBAT and PBS allowed the formation of co-continuously phases to improve the mechanical properties. The bio-composite TPRH36/24 possessed good mechanical properties such as tensile strength (14.27 MPa), Young’s modulus (200.43 MPa), and elongation at break (12.99%), which is adequate for the manufacturing of molded products such as a tray, lunch box, and straw. Finally, it achieved a 92% mass loss after six months, evidencing itself as a biodegradable material. The test results from this study indicated an accomplishment in the fabrication of cost-efficient biodegradable polymer using waste fillers, which has tremendous potential for practical use in various industrial applications.

## Figures and Tables

**Table 1:** The summary of biodegradable polymer/rice husk composites with their respective composition, plasticizer, and mechanical properties.

Material	Ratio	Compatibilizer	Tensile Strength (MPa)	Young’s Modulus (MPa)	Elongation at Break (%)	Reference
PP/rice husk	60/40	-	21.5	-	4.7	[18]
PBAT/rice husk silica	70/30	-	14.5	54	820	[19]
PLA/AT rice husk	75/25	-	7.35	-	0.99	[20]
PP/rice husk	90/10	-	32.50	1944	-	[23]
	80/20	-	31.78	2209	-	[23]
	70/30	-	31.42	2540	-	[23]
PP/Rice husk	70/30	-	24	2100	-	[24]
PP/rice husk	70/30	-	29.1	2000	-	[25]
PP/rice husk	50/50	-	17.76	2134	-	[26]
PP/rice husk	60/40	struktol	20	5000	-	[27]
PLA/rice husk	100/0 95/5	-	0.22 0.24	0.2 2	45 7	[28]
						[28]

AT: Alkaline treated, PP: polypropylene, PBAT: poly butylenes adipate-Co-terephthalate, PLA: polylactic acid.

**Table 2:** The compositions of the PBAT/PBS/TPS and PBAT/PBS/TPRH for the fabrication of biodegradable polymers.

Designation	Composition	Parts
PBS	PBS	100
PBAT	PBAT	100
TPRH48/12	PBAT/PBS/TPRH/CS/MA/DCP	48/12/40/0.5/2/0.4
TPS48/12	PBAT/PBS/TPS/CS/MA/DCP	48/12/40/0.5/2/0.4
TPRH 36/24	PBAT/PBS/TPRH/CS/MA/DCP	36/24/40/0.5/2/0.4
TPS36/24	PBAT/PBS/TPS/CS/MA/DCP	36/24/40/0.5/2/0.4
Commercial PBAT	-	-

CS: calcium stearate, MA: maleic anhydride, DCP: Dicumyl peroxide, PBS: polybutylene succinate, TPS: thermoplastic starch, TPRH: thermoplastic rice husk.

**Table 3:** Tensile strength, Young’s modulus, and elongation at break for PBS/PBAT/TPS and PBS/PBAT/TPRH composites.

Sample Code	Tensile Strength (MPa)	Young’s Modulus (MPa)	Elongation at Break (%)
PBS	30.63 ± 2.4	166.23 ± 2.71	547.45 ± 21.61
PBAT	38.99 ± 7.25	16.1 ± 2.63	1421.93 ± 123
TPRH48/12	12.43 ± 0.48	150.34 ± 4.43	24.70 ± 2.53
TPS48/12	10.42 ± 0.52	134.38 ± 3.63	28.62 ± 3.16
TPRH36/24	14.27 ± 1.13	200.43 ± 14.73	12.99 ± 2.34
TPS36/24	14.21 ± 0.81	199.49 ± 9.03	15.39 ± 0.98
Commercial PBAT	10.07 ± 0.99	55.84 ± 2.77	716.95 ± 125.44

**Table 4:** Thermal properties of bare PBAT, bare PBS, PBAT/PBS/RH blends, and PBAT/PBS/TPS blends.

Code	T_m_ (°C)	Enthalpy of Melting of 100% Crystalline, ∆H_m100_ (J/g)	X_c_ (%)
PBS	114.04	70.62	64.03
PBAT	121.68	9.76	8.56
TPRH48/12	110.29	8.49	15.52
TPS48/12	111.27	7.92	14.47
TPRH36/24	111.38	14.78	36.01
TPS36/24	111.72	17.69	43.10
Commercial PBAT	119.77	1.4	1.75

**Table 5:** Mass loss percentage of PBS/PBAT blends after soil burial test for six months.

Sample Code	Mass Loss Percentage (%)			
	0 month	2 months	4 months	6 months
PBS	0	4.19 ± 0.29	6.54 ± 0.10	8.90 ± 0.06
PBAT	0	6.23 ± 0.31	6.82 ± 0.11	9.20 ± 0.05
TPRH48/12	0	20.94 ± 0.14	64.56 ± 0.21	79.78 ± 0.10
TPS48/12	0	48.74 ± 0.21	83.30 ± 0.27	86.88 ± 0.19
TPRH36/24	0	31.52 ± 0.30	88.91 ± 0.08	92.00 ± 0.08
TPS36/24	0	53.36 ± 0.17	92.07 ± 0.08	97.06 ± 0.03
Commercial PBAT	0	14.27 ± 0.15	18.16 ± 0.19	32.51 ± 0.17

## Data Availability

Not applicable.
